# Event-Related Potentials Dissociate Effects of Salience and Space in Biased Competition for Visual Representation

**DOI:** 10.1371/journal.pone.0012677

**Published:** 2010-09-16

**Authors:** Matthew R. Hilimire, Jeffrey R. W. Mounts, Nathan A. Parks, Paul M. Corballis

**Affiliations:** 1 School of Psychology, Georgia Institute of Technology, Atlanta, Georgia, United States of America; 2 Psychology Department, State University of New York at Geneseo, Geneseo, New York, United States of America; 3 Beckman Institute, University of Illinois at Urbana-Champaign, Urbana-Champaign, Illinois, United States of America; Macquarie University, Australia

## Abstract

**Background:**

Selective visual attention is the process by which the visual system enhances behaviorally relevant stimuli and filters out others. Visual attention is thought to operate through a cortical mechanism known as biased competition. Representations of stimuli within cortical visual areas compete such that they mutually suppress each others' neural response. Competition increases with stimulus proximity and can be biased in favor of one stimulus (over another) as a function of stimulus significance, salience, or expectancy. Though there is considerable evidence of biased competition within the human visual system, the dynamics of the process remain unknown.

**Methodology/Principal Findings:**

Here, we used scalp-recorded electroencephalography (EEG) to examine neural correlates of biased competition in the human visual system. In two experiments, subjects performed a task requiring them to either simultaneously identify two targets (Experiment 1) or discriminate one target while ignoring a decoy (Experiment 2). Competition was manipulated by altering the spatial separation between target(s) and/or decoy. Both experimental tasks should induce competition between stimuli. However, only the task of Experiment 2 should invoke a strong bias in favor of the target (over the decoy). The amplitude of two lateralized components of the event-related potential, the N2pc and Ptc, mirrored these predictions. N2pc amplitude increased with increasing stimulus separation in Experiments 1 and 2. However, Ptc amplitude varied only in Experiment 2, becoming more positive with decreased spatial separation.

**Conclusions/Significance:**

These results suggest that N2pc and Ptc components may index distinct processes of biased competition—N2pc reflecting visual competitive interactions and Ptc reflecting a bias in processing necessary to individuate task-relevant stimuli.

## Introduction

A typical visual scene is cluttered with many objects, yet we are only subjectively aware of a small subset of those objects at any given time. Even though the optical information about the scene impinges on our retina and is transduced by the photoreceptors, only a small amount of the available information is processed to the level of consciousness. There is a great deal of evidence that the selection of visual information for higher visual processing is not random; objects or stimuli that are relevant to current goals are more likely to be represented than irrelevant or distracting information. Our ability to selectively process some objects at the expense of others is known as visual selective attention. It is a major goal of researchers studying visual attention to understand why the visual system is limited in capacity and cannot represent every object in the visual scene simultaneously. It is likewise important to understand how goal-relevant information is selected – and how irrelevant or distracting information is suppressed – when many objects are present in a scene.

One influential theory of visual selective attention, Desimone and Duncan's [1] biased competition theory, holds that the limited capacity of the visual system is a necessary consequence of the architecture of the object-selective regions of the extrastriate visual cortex. This theory is based on the observation that each visual neuron in the extrastriate cortex seems to be able to optimally represent only one object at a time. When multiple objects are present in the receptive field (RF; the region of the visual field to which a visual neuron responds) of a visual neuron, they compete to control the response of that neuron. Eventually, only one of the stimuli is represented by that neuron. This competition for representation, coupled with the observation that neurons in the extrastriate visual cortex often have large RFs that overlap with those of many other neurons, necessarily imposes a strict limit on the amount of visual information that can be represented by the visual system as a whole [1]–[2]. In addition, because this competition occurs at the level of the RF, competition for representation is spatially-mediated such that competition increases as two objects get closer together, as this increases the proportion of visual system neurons whose receptive fields are stimulated by both objects.

While the biased competition model is derived primarily from single-cell neurophysiology studies conducted in non-human primates, there is mounting evidence from both psychophysical and electrophysiological studies in humans for spatially-mediated competitive interactions between visual stimuli. Behavioral manifestations of these competitive interactions have been observed in a number of different experimental paradigms [3]–[7]. In one of the first studies reporting behavioral evidence of competitive interactions, Bahcall and Kowler [8] utilized a divided attention task in which they asked observers to report the identities of two spatially cued letters from a circular array. They found that identification accuracy deteriorated as the spatial proximity of the target letters decreased, consistent with the idea that spatial proximity increases the proportion of RFs shared by the target letters, resulting in greater competition between them.

Electrophysiological investigations of visual selective attention have revealed the existence of a component of the event-related potential (ERP), termed the N2pc, that is generally believed to reflect neural processes related to the attentional selection of objects [9]–[10]. The N2pc is typically observed approximately 200–250 ms post-stimulus and is defined as an enhanced negative voltage at posterior electrodes contralateral to attended stimuli compared to ipsilateral electrodes [11]–[12]. The N2pc may reflect processes related to both detecting target related features [11] and suppressing distractor processing [9]. Additionally, the neural generators of the N2pc component have been localized to extrastriate visual areas [9], [12].

In a previous study, we utilized the N2pc component of the ERP to investigate competition among visual stimuli for representation [13]. We manipulated competition in a target/decoy paradigm adapted from Mounts, McCarley, and Terech [4]. Two colored items were embedded in an array of gray filler items and we varied the separation between the colored items, one the target and the other a decoy. Participants responded to the orientation of the target. We found that interference was greatest from the decoy when the target and decoy were adjacent and the interference decreased as the spatial separation between the target and decoy increased. Specifically, N2pc amplitude was smallest when the target and decoy were adjacent and was larger as the distance between the two attended items increased. We concluded that the reduced N2pc amplitude may indicate degraded target selection processes due to increased competition for representation between the target and attentionally salient decoy when they are spatially proximal. To ensure that these N2pc differences reflected attentionally-mediated competition and were not a consequence of sensory interactions, we also had participants perform a localization task on the same stimulus configurations. Participants responded whether the two colored objects appeared to the left or right of fixation and the results showed that N2pc amplitude did not vary with the separation between the two colored objects. Thus, the N2pc results did not reflect sensory interactions but rather localized competition when the target had to be individuated and identified.

We also documented a subsequent component, termed the Ptc, as a component that potentially indexes additional processing that individuates or isolates one of the objects (i.e., the target) after it is identified. The Ptc starts approximately 290 ms post-stimulus and persists until approximately 340 ms and manifests as a positivity contralateral to the attended items. The Ptc component was distributed more towards temporal electrodes compared to the more posterior N2pc component. In contrast to the N2pc results, Ptc amplitude was largest (i.e., more positive) when the target and decoy were adjacent and was smaller as the distance between the two attended items increased. These results suggest that Ptc amplitude may be influenced by the amount of additional processing that is necessary to overcome the spatially-mediated competition for representation. Because the Ptc occurs after the N2pc component, it is likely that the Ptc reflects processing subsequent to target identification and may reflect processes used to individuate or isolate the target once it is identified.

In the current study, we conducted two experiments designed to further elucidate the relationship between the N2pc and Ptc components and neural competitive interactions. Our main goal was to functionally dissociate the processes reflected by these two components. Previously, we argued that the N2pc and Ptc are distinct ERP components that reflect different aspects of biased competition [13]. The N2pc and Ptc occur at different times (the N2pc occurs before the Ptc), they have different scalp distributions (the N2pc is more posterior and the Ptc is more temporal), and they showed opposite effects (the N2pc was attenuated when the target and decoy were nearby but the Ptc was potentiated). However, we could not entirely rule out the possibility that the Ptc may simply reflect late N2pc activity. It is possible, for example, that when the N2pc is large in amplitude, it persists into the Ptc time window and results in decreased Ptc amplitude. In this case, the Ptc component should covary with the same manipulations as the N2pc, but with the opposite polarity (as we reported). If, however, the Ptc is in fact a distinct component reflecting different processing, the N2pc and Ptc should be dissociable. Specifically, it should be possible to introduce manipulations that will affect N2pc amplitude without changing Ptc amplitude, and the N2pc and Ptc components should be shown to be sensitive to different manipulations. Our goal in this study was to determine whether manipulations of task (Experiment 1) and stimulus salience (Experiment 2) would have differential influences on N2pc and Ptc amplitudes.

### Experiment 1

**Figure 1:**
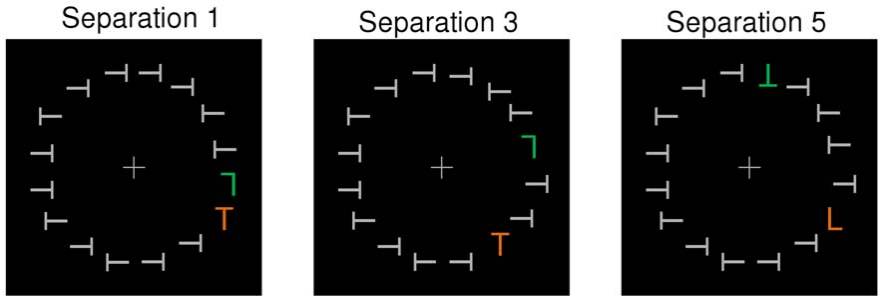
Examples of the stimulus displays used in Experiment 1. The two colored items (orange and green) were the targets and participants judged whether they were the same or different letters. The examples show displays where participants would respond “different.” Target-target separation was varied in three levels: Separation 1, Separation 3, and Separation 5.

Here we sought to demonstrate that it is possible to influence the N2pc in the absence of Ptc modulation, which should be possible if these components reflect distinct mechanisms of visual selective attention. In order to do this, we selected a task that would invoke spatially-mediated competition without the necessity of resolving the competition in favor of one of the two objects. In the target/decoy paradigm used by Hilimire and colleagues [13], it is optimal to select one of the colored items (the target) and minimize the representation of the non-selected item (the decoy). We reasoned that this required additional processing to individuate or isolate the target, and that this additional processing was the source of the Ptc component. In light of this, we changed the task to a same-different procedure, which should eliminate this additional stage in processing. We used similar displays to those used by Hilimire and colleagues [13] but adapted for the same/different task (see **Figure 1**). Participants had to determine whether a green and an orange letter were the same (i.e., both Ts or both Ls) or different (i.e., one T and one L). In this task, it is necessary for the participant to represent both items in their visual system to perform the task. Therefore, there should not be any additional processing to individuate or isolate one target, as both items are targets. Based on this logic, we predicted that in the same/different task we would observe spatial modulation of the N2pc but no Ptc effects. Specifically, we predicted that N2pc amplitude would decrease as the separation between the two targets is reduced. We suggest that the diminished N2pc amplitude reflects degraded target selection due to spatially-mediated competition. In addition, we predicted that Ptc amplitude will not vary with the separation between targets.

### Experiment 2

In Experiment 2, we explored the relationship between the N2pc and Ptc by attempting to further dissociate the components. We returned to the target/decoy paradigm used by Hilimire and colleagues [13] and we varied the separation and relative salience between two attended objects. Participants performed an orientation discrimination task where they responded to the orientation of a target (a green or orange T) while ignoring a decoy (an L of the other color) among gray distractors. Because the color of the target was chosen randomly on each trial, participants needed to attend to both colored items to perform the task effectively. In addition, we manipulated the relative salience of the target and decoy by changing the saturation of their colors (making them less distinct from the gray filler items).

We hypothesized that the N2pc component would be sensitive to both the target-decoy separation manipulation and the relative salience manipulation. Specifically, when the target and decoy are close together, N2pc amplitude will be reduced compared to when they are farther apart. A reduction in N2pc amplitude when the target and decoy are near each is consistent with the idea that spatially-mediated competition for representation degrades target selection processes. In addition, we predicted that N2pc amplitude would increase when the decoy is relatively salient compared to when the target is relatively salient. When the decoy is relatively salient, it is likely that participants will first select the salient decoy and then subsequently select the target because the competition for representation is initially biased towards the most salient item in the display [1], [14]–[15]. N2pc amplitude has been shown to be sensitive to shifts of attention between a highly salient distractor and a less salient target [16]–[17]. Thus, we hypothesized that N2pc amplitude would be sensitive to the any shifts of attention between the decoy and the target. Specifically, we predicted that N2pc should be larger when the decoy is relatively salient which would be consistent with the idea that first the salient decoy is selected with a subsequent shift to the target. In contrast, we predicted that N2pc amplitude would be smaller when the target is more salient which would be consistent with the idea that the target is selected initially and there is no additional shift of attention necessary.

Regarding the Ptc component, we hypothesized that it would only be sensitive to the target-decoy separation manipulation and not the relative salience manipulation. We expected that greater additional processing would be necessary when the target and decoy are near each other. Specifically, when the target and decoy are near each other, the Ptc component may reflect processes used to individuate or isolate the target once it is identified. However, when the two objects are farther apart, there is minimal competition for representation which reduces the need for these target individuation processes. Therefore, we hypothesized that Ptc amplitude would be greatest (i.e., most positive in amplitude) when the target and decoy were near each other compared to when they were far apart. The Ptc component should not vary with the relative salience manipulation because the Ptc probably reflects processes that occur after the target is identified. By the time the Ptc component is evident, participants should have already completed any shifts of attention between the decoy and target and are implementing processes to individuate or isolate the target. If the N2pc is sensitive to both manipulations but the Ptc is only sensitive to the distance manipulation, this will provide further evidence that the two components are distinct and reflect different processes related to biased competition.

## Methods

### Experiment 1

#### Participants

The participants were 15 undergraduate students at Georgia Institute of Technology that participated for course credit. Twelve (*M* = 20.3 years, *S.D.* = 3.2 years, 5 women) of these participants were included in the analysis (see below for exclusion criteria). All participants provided informed consent and all research was approved by the institutional review board at Georgia Institute of Technology. In addition, all participants reported normal or corrected-to-normal vision.

#### Stimuli

The stimulus displays consisted of 16 letters (Ts and Ls; 1.2°×1.2°) arranged in equal intervals around an imaginary circle with a radius of 6° of visual angle centered on fixation (grey cross; 1.2°×1.2°; see **Figure 1**). The displays were presented on a uniform black background. Fourteen of the letters were ‘filler’ items which were grey, footlambert (fL) = 23.05, Ts that were randomly rotated 90° to the left or right. The remaining two letters were the targets. The two targets were upright or inverted Ts and Ls that were colored either orange (x = 0.45, y = 0.45, fL = 21.44) or green (x = 0.28, y = 0.54, fL = 21.23). On each trial, one target was orange and the other target was green. Participants had to attend to both colored items in order to perform the task. The two targets were separated by either one position (adjacent), three positions (two intervening fillers), or five positions (four intervening fillers). This yielded three levels of target-target separation corresponding to angular distances of 22.5° (separation 1), 67.5° (separation 3), and 112.5° (separation 5). In degrees of visual angle, the center-to-center distances were approximately 2° (separation 1), 6° (separation 3), and 10° (separation 5). The two targets always appeared in the same visual hemifield (i.e., both is the left or right visual field), and occurred equally often at each of the 16 possible stimulus locations.

#### Procedure

Participants were seated in a darkened, sound-attenuating booth. Experimental stimuli were presented on a 21-inch CRT monitor positioned 57 cm from the participant with viewing distance maintained through the use of a chinrest. Each trial began with a grey fixation cross on a black background that remained visible for a random interval between 500 and 1500 ms. The stimulus array was then flashed for 200 ms and a blank screen remained present until a response was given. The participants were instructed to report whether the two targets were the same letter or different letters as quickly as possible while maintaining approximately 90% accuracy. Responses were given using the number pad of a standard keyboard using the right hand (‘1’ for same with right index finger, ‘2’ for different with right middle finger). Incorrect responses were signaled by an ‘X’ displayed at the center of the screen. Participants completed 24 practice trials followed by 24 blocks of 48 experimental trials each for a total of 1152 trials. The order of trials was randomized within each block.

#### Electrophysiological Recording and Analyses

Electrophysiological data were recorded using a Biosemi ActiveTwo amplifier system (Amsterdam, Netherlands). Scalp potentials were recorded from 32 electrodes: FP1/FP2, AF3/4, FC1/2, FC5/6, F7/8, F3/4, Fz, C3/4, Cz, CP1/2, CP5/6, P7/8, PO3/4, P3/4, Pz, T7/8, O1/2, and Oz. Two additional electrodes were placed on the mastoids. Finally, the ActiveTwo system requires the placement of two additional electrodes: common mode sense (CMS) and driven right leg (DRL). The electroencephalogram (EEG) was digitized at 1024 Hz and was acquired with respect to the CMS electrode.

EEG data were analyzed using BrainVision Analyzer (Brain Products, Gilching, Germany). Offline, all channels were re-referenced to the algebraic average of the left and right mastoids. Electrooculogram (EOG) was calculated offline as the difference between electrodes positioned above and below the left eye and on the outer canthi of each eye for VEOG and HEOG, respectively. Continuous EEG was digitally band-pass filtered from 0.1 to 30 Hz using a zero phase-shift Butterworth filter (12 dB/oct). EEG was segmented into 900 ms segments beginning 200 ms pre-stimulus and continuing 700 ms post-stimulus. Segments were then baseline corrected by setting the average of the 200 ms pre-stimulus baseline to zero. Segments containing activity greater than ±80 µV in the scalp and VEOG channels were considered artifacts and rejected. Additionally, we used a two-step procedure to exclude eye-movements.

First, activity greater than ±50 µV in the HEOG channel were considered artifacts and rejected. Next, participants' averages were formed for right and left visual field targets separately. Participants were excluded if average HEOG activity exceeded ±5 µV (3 participants were excluded and the resulting grand average HEOG activity of the remaining 12 participants did not exceed ±3.5 µV). This artifact rejection procedure ensured that no systematic eye-movements over 0.3° were included in the data. Participant averages for each level of target-target separation were formed separately for ipsilateral and contralateral electrodes. Grand average waveforms were formed from the subject averages in each condition.

The N2pc component was quantified as the mean amplitude in a 50 ms window (200–250 ms) and the Ptc component was quantified as the mean amplitude in a 50 ms window (280–330 ms) of the contralateral/ipsilateral difference waveforms separately for each level of target-target separation at electrodes P7/8 and PO3/4. This time window was chosen based on the peaks in the grand average waveform across all conditions. The mean amplitudes were then tested using a RANOVA with target-target separation as a within-subjects factor.

### Experiment 2

The methodology was similar to Experiment 1 with the following exceptions.

#### Participants

There were 15 participants and 13 (*M* = 20.4 years, *S.D.* = 1.2 years, 6 women) of these participants were included in the analysis based on the exclusion criteria explained in Experiment 1. None of the participants participated in Experiment 1.

#### Stimuli

The two colored letters were the ‘target’ and the ‘decoy’. The target was an upright or inverted T that was colored either orange or green. The color of the target was chosen randomly on each trial. The decoy was an upright or inverted L that was also colored either orange or green but was the opposite color of the target. Because the color of the target was selected at random, participants had to attend to both colored items in order to perform the task. The target and decoy were either near each other (adjacent) or far apart (four intervening fillers). This yielded two levels of target-decoy separation corresponding to angular distances of 22.5° (near) and 112.5° (far). In degrees of visual angle, the center-to-center distances were approximately 2° (near) and 10° (far). The relative salience of the target and decoy was also manipulated by adjusting the color saturation of the target or decoy such that it was highly salient relative to filler items (100% saturation) or was of considerably reduced salience relative to the filler items (approximately 50% saturation). The color values and luminance were as follows: saturated orange (x = 0.45, y = 0.45, fL = 21.44); desaturated orange (x = 0.33, y = 0.35, fL = 21.85); saturated green (x = 0.28, y = 0.54, fL = 21.23); desaturated green (x = 0.28, y = 0.39, fL = 21.72). Thus, luminance was approximately equal across all colors used in the experiment to control for sensory differences. On half the trials, the decoy was less salient than the target (target salient condition) and on the other half, the target was less salient (decoy salient condition).

#### Procedure

The participants were instructed to report the orientation of the target and responses were given using the number pad of a standard keyboard using the right hand (‘1’ for an inverted T with right index finger, ‘2’ for an upright T with right middle finger).

#### Electrophysiological Recording and Analyses

Two participants were excluded and the resulting grand average HEOG activity of the remaining 13 participants did not exceed ±3.5 µV. Participant averages for each level of target-decoy separation and relative salience were formed separately for ipsilateral and contralateral electrodes.

## Results

### Experiment 1

#### Behavioral Data

**Figure 2:**
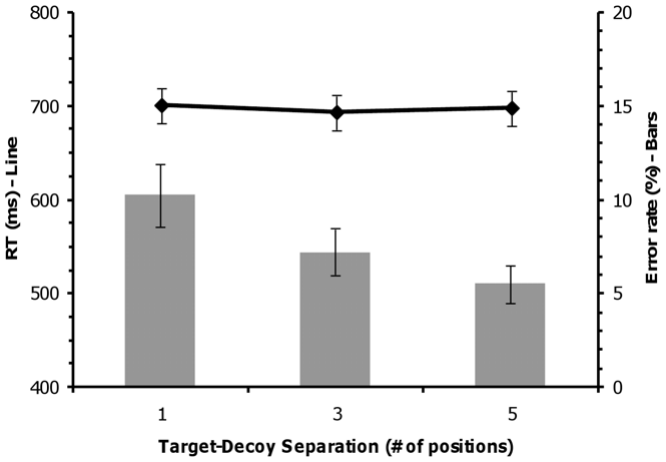
Behavioral results for Experiment 1. Mean reaction time (line) and error rates (bars) as a function of target-target separation for the same/different task in Experiment 1. Error bars are standard error. Note that participants committed more errors as the targets get closer together.

Reaction time and error rates were tested using repeated measures analyses of variance (RANOVAs; all RANOVAs were Huynh-Feldt corrected where appropriate) with target-target separation as a within-subjects factor. Statistically reliable effects were elucidated using linear contrasts and pair-wise comparisons. The error rate data (see **Figure 2**) showed a significant effect of target-target separation, *F*(2,22) = 8.00, *p*<0.05, ε = 0.73, η_p_^2^ = 0.42. A significant linear trend was present in the error rate data, *t*(11) = 2.41, *p*<0.05, indicating that participants committed more errors with decreasing target-target separation. Pair-wise comparisons revealed significant differences in error rate between separation 1 and separation 3, *t*(11) = 2.42, *p*<0.05 and between separation 1 and separation 5, *t*(11) = 3.41, *p*<0.05, but failed to reach significance between separation 3 and separation 5, *t*(11) = 1.70, *p* = 0.116. The reaction time data (see **Figure 2**) did not show a statistically significant effect of target-target separation, *F*(2,22) = 1.68, *p* = 0.22, ε = 0.63, η_p_^2^ = 0.13. The behavioral results indicate that attentional competition occurred such that participants were less accurate in their performance of the same-different task when the two targets were close to each other.

#### N2pc Component

**Figure 3:**
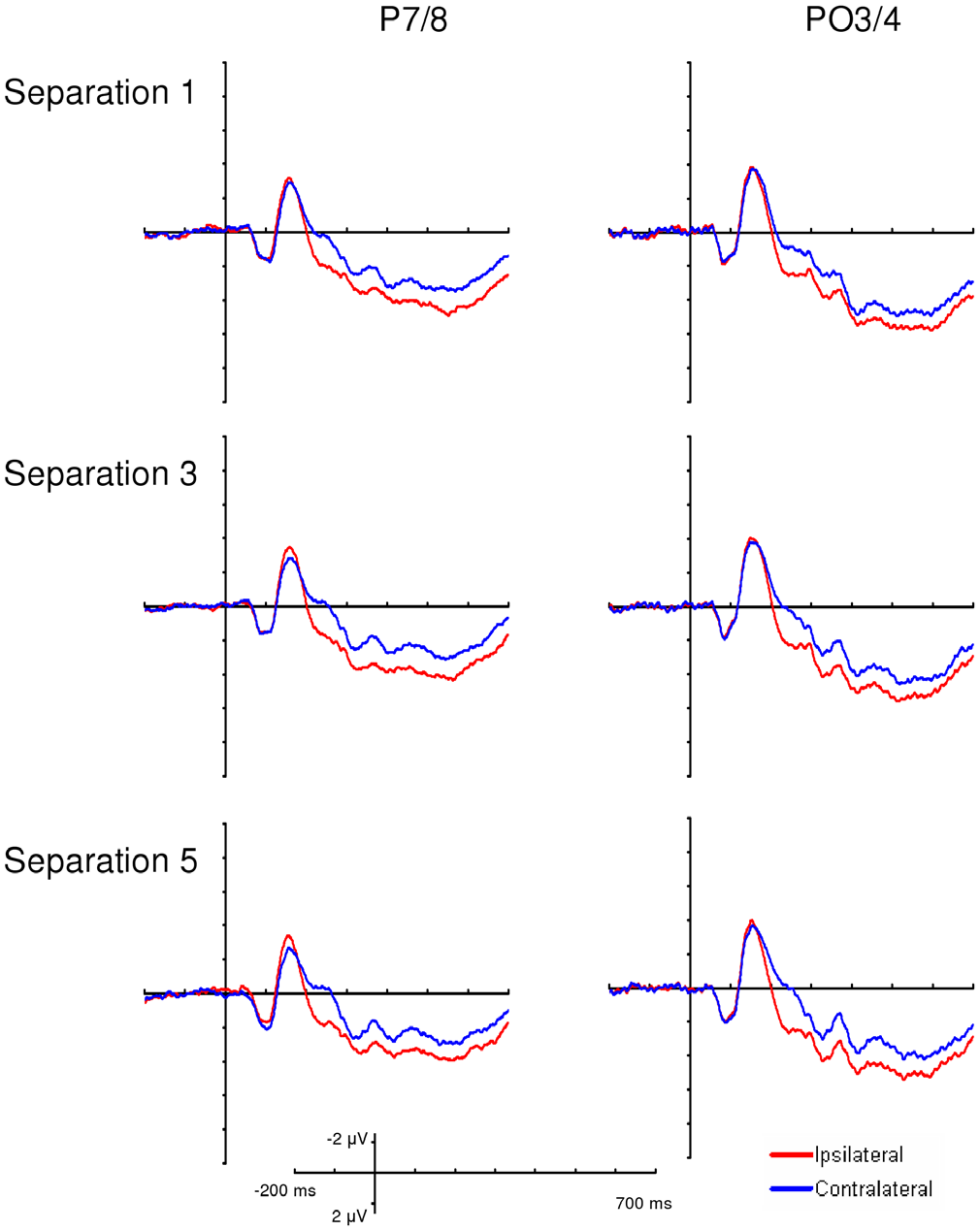
Ipsilateral and contralateral waveforms from Experiment 1. Displayed are the ipsilateral and contralateral grand average waveforms for electrodes P7/8 and PO3/4 at each level of target-target separation from Experiment 1. Note that N2pc amplitude increases with increasing target-decoy separation and Ptc amplitude does not vary with target-target separation.

**Figure 4:**
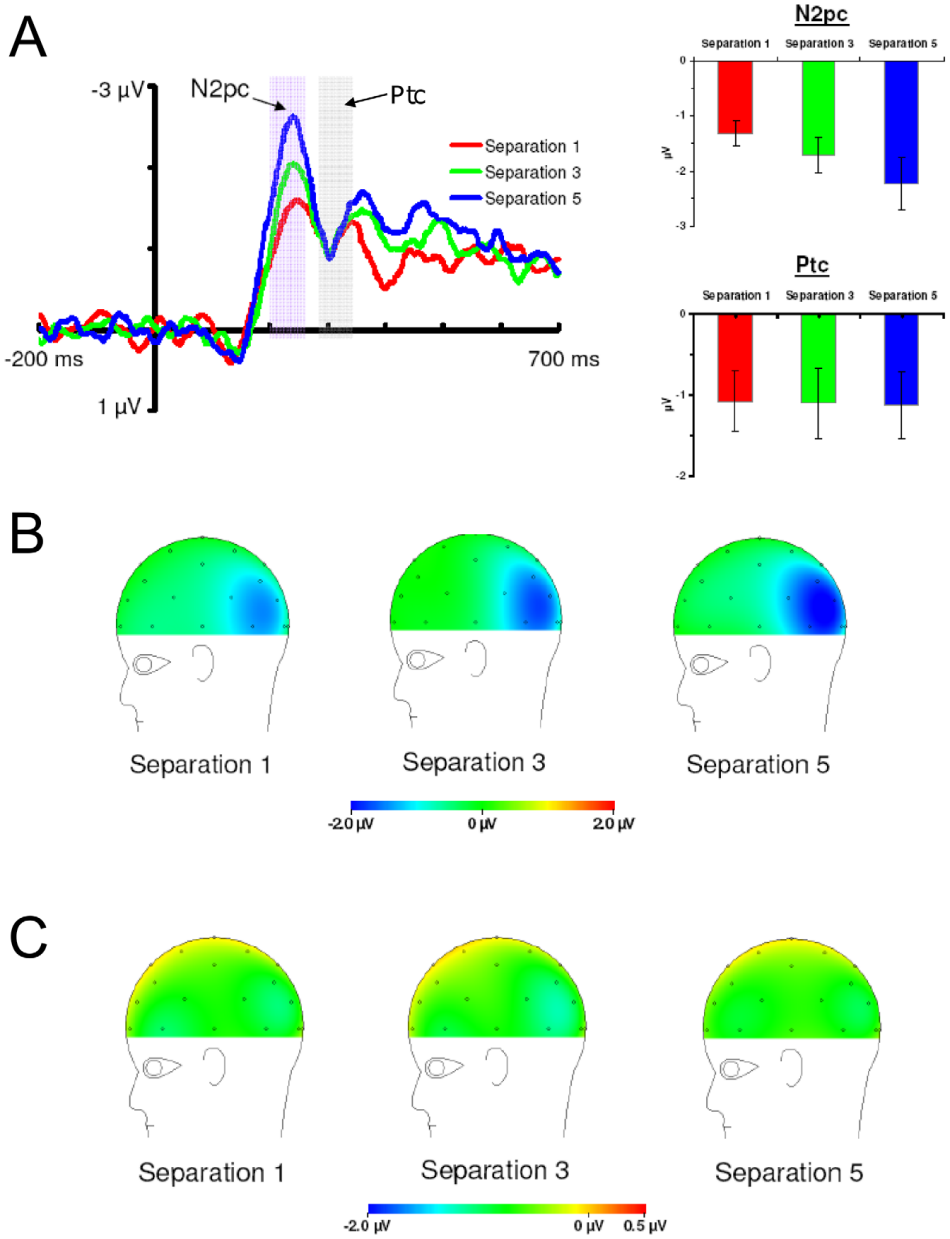
Difference waveforms and scalp distributions from Experiment 1. (a) Left: Ipsilateral/contralateral difference waveforms at electrodes PO3/4 from Experiment 1. These were calculated by subtracting the ipsilateral waveforms from the contralateral waveforms plotted in Figure 3. These difference waveforms allow N2pc and Ptc amplitude to be compared across levels of target-target separation. Right: N2pc and Ptc amplitudes as a function of target-target separation. Note that N2pc amplitude increases with increasing target-target separation and Ptc amplitude does not vary with target-decoy separation. Error bars are standard error. (b) Scalp distribution of the N2pc. (c) Scalp distribution of the Ptc.

The amplitude of the N2pc component is larger when the two targets are far apart and smaller when the two targets are near each other (see **Figures 3** **&** **4a**). **Figure 4b** shows the scalp distributions of the N2pc. Note that the N2pc is distributed around posterior electrodes contralateral to the attended items and that N2pc amplitude varies with target-target separation.

The N2pc data showed a significant effect of target-target separation at electrodes PO3/4, *F*(2,22) = 6.73, *p*<0.05, ε = 0.69, η_p_^2^ = 0.38. A significant linear trend was present in the N2pc data at PO3/4, *t*(11) = 7.86, *p*<0.05, such that N2pc amplitude was largest when the two targets were farthest apart and diminished as the distance between the two targets decreased. Pair-wise comparisons revealed significant differences in N2pc amplitude at PO3/4 between separation 1 and separation 3, *t*(11) = 2.40, *p*<0.05, between separation 1 and separation 5, *t*(11) = 2.80, *p*<0.05, and between separation 3 and separation 5, *t*(11) = 2.23, *p*<0.05. The N2pc data did not show a significant effect of target-target separation at electrodes P7/8, *F*(2,22)<1, η_p_^2^ = 0.06. These results are consistent with the idea that the reduction in N2pc amplitude is indexing degraded target selection processes due to spatially-mediated competition between the two targets.

We conducted an additional analysis to examine an alternative explanation of our results based on the positioning of the two targets. It is known that the N2pc varies in amplitude between the upper and lower visual fields such that N2pc amplitude is greater when a salient item is presented in the lower visual field compared to the upper visual field (e.g., Luck et al., 1997). In our Separation 1 condition, the two targets could appear both in the upper visual field or both in the lower visual field. However, in our Separation 3 and Separation 5 conditions, it was possible for one target to appear in the lower visual field while the other appeared in the upper visual field. A possible alternative explanation of our results is that the N2pc amplitude differences observed merely reflect an initial bias towards lower visual field salient objects. To explore this possible alternative explanation, we compared N2pc amplitude for Separation 1 (both targets in lower visual field) vs. Separation 3 and 5 (one target in the lower visual field). If our results are due only to visual field differences, we would expect similar N2pc amplitudes when at least one target is in the lower visual field. Thus, the N2pc amplitude in the Separation 1 condition with both targets in the lower visual field should be equivalent to the N2pc amplitude in the Separation 3 and Separation 5 conditions when one target is in the lower visual field. If our results are due to the distance between the two targets, we would expect a larger N2pc when the targets are far apart (Separation 3 and Separation 5 conditions with one target in lower visual field) compared to when they are near each other (Separation 1 condition with both targets in the lower visual field). Using one-tailed, paired-samples *t*-tests at electrodes P7/8 and PO3/4, we compared the average N2pc amplitude of trials with targets in the Separation 3 and Separation 5 conditions with one target in the lower visual field to the N2pc amplitude of trials in the Separation 1 condition with both targets in the lower visual field. N2pc amplitude was greater for the Separation 3 and Separation 5 condition compared to the Separation 1 condition at electrodes PO3/4, *t*(11) = 2.97, *p*<0.05, and marginally greater at P7/8, *t*(11) = 1.93, *p* = 0.079. Thus, this analysis supports the idea that the N2pc reduction at Separation 1 compared to Separation 3 and Separation 5 was due to the fact that the two targets were near each other in the Separation 1 condition and farther apart in the Separation 3 and Separation 5 conditions.

#### Ptc Component

Ptc amplitude does not vary with target-target separation (see **Figures 3** **&** **4a**). Additionally, the scalp distributions from the 280–330 ms time window show no differences based on target-target separation (see **Figure 4c**) and thus the effect of target-target separation was not statistically significant at electrodes P7/8, *F*(2,22) = 1.00, *p* = 0.38, ε = 0.99, η_p_^2^ = 0.08, or PO3/4, *F*(2,22)<1, η_p_^2^ = 0.00. Here we find no evidence that the Ptc component varied with target-target separation.

### Experiment 2

#### Behavioral Data

**Figure 5:**
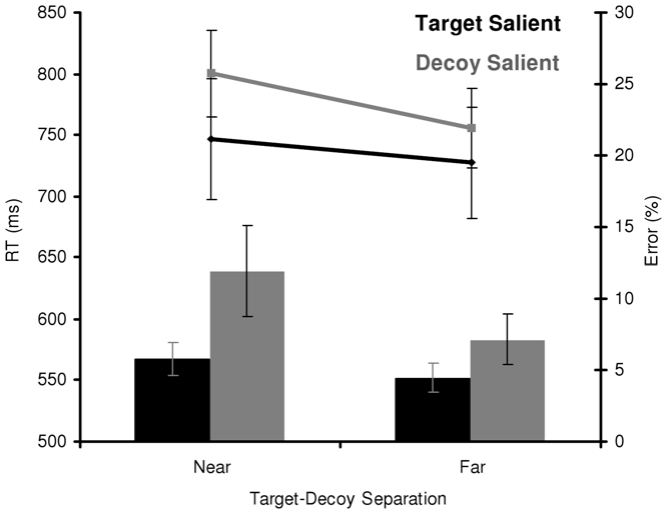
Behavioral results for Experiment 2. Mean reaction time (line) and error rates (bars) as a function of target-decoy separation and relative salience for the orientation discrimination task in Experiment 2. Error bars are standard error. Note that participants were slower and committed more errors as the target and decoy get closer together. Additionally, participants were slower and committed more errors when the decoy was relatively more salient than the target but only when the target and decoy were near each other.

Reaction time and error rates were tested using RANOVAs with target-decoy separation (near or far) and relative salience (target salient or decoy salient) as within-subjects factors. Statistically reliable interactions were elucidated by examining the simple main effects using pair-wise comparisons. The reaction time data (see **Figure 5**) showed a main effect of target-decoy separation, *F*(1,12) = 32.40, *p*<0.05, η_p_^2^ = 0.73, indicating that participants were faster to respond to the orientation of the target when the target and decoy were far apart compared to when they were near each other. There was also a marginally significant main effect of relative salience, *F*(1,12) = 3.54, *p* = 0.08, η_p_^2^ = 0.23, indicating that participants were faster to respond when the target was more salient than the decoy. However, the main effects are qualified by an interaction between target-decoy separation and relative salience, *F*(1,12) = 7.04, *p*<0.05, η_p_^2^ = 0.37. To examine this interaction, simple main effects were analyzed using two-tailed, paired-samples *t*-tests. Both when the target was salient and when the decoy was salient, participants were faster to respond when the target and decoy were far apart compared to near each other, *t*(12) = 3.03, *p*<0.05 and *t*(12) = 5.51, *p*<0.05, respectively. When the target and decoy were near each other, participants were faster to respond when the target was salient compared to when the decoy was salient, *t*(12) = 2.20, *p*<0.05. In contrast, when the target and decoy were far apart, the effect of relative salience was not statistically significant, *t*(12) = 1.42, *p* = 0.18.

The error rate data (see **Figure 5**) showed a main effect of target-decoy separation, *F*(1,12) = 9.94, *p*<0.05, η_p_^2^ = 0.45, indicating that participants were more accurate in responding to the orientation of the target when the target and decoy were far apart compared to when they were near each other. The main effect of relative salience was not statistically significant, *F*(1,12) = 2.95, *p* = 0.11, η_p_^2^ = 0.20. The main effect of target-decoy separation was qualified by a marginally significant interaction between target-decoy separation and relative salience, *F*(1,12) = 3.89, *p* = 0.07, η_p_^2^ = 0.25.

#### N2pc Component

**Figure 6:**
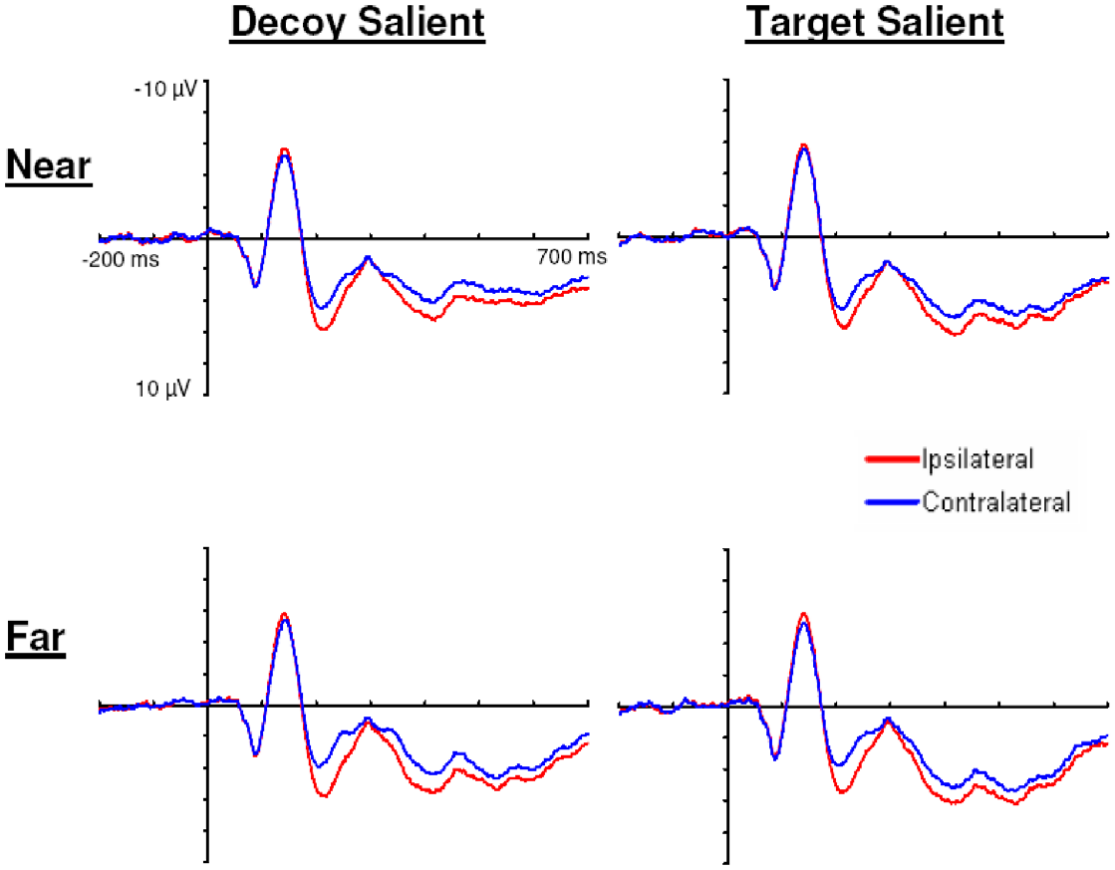
Ipsilateral and contralateral waveforms from Experiment 2. Displayed are the ipsilateral and contralateral grand average waveforms for electrodes PO3/4 at each level of target-decoy separation and relative salience in Experiment 2.

**Figure 7:**
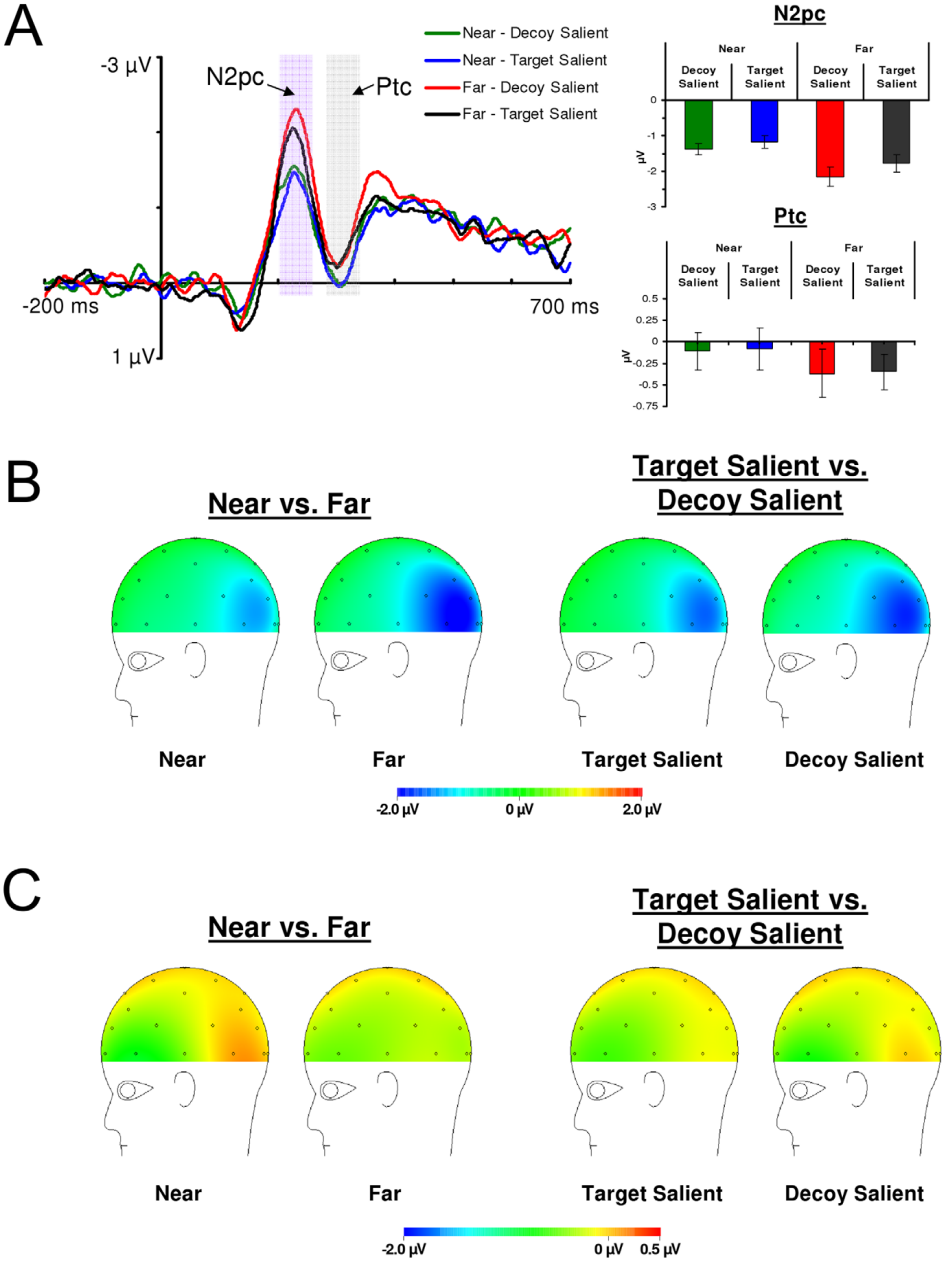
Difference waveforms and scalp distributions from Experiment 2. (a) Left: Ipsilateral/contralateral difference waveforms at electrodes PO3/4 for Experiment 2. These were calculated by subtracting the ipsilateral waveforms from the contralteral waveforms plotted in Figure 6. These difference waveforms allow N2pc and Ptc amplitudes to be compared across levels of target-decoy separation and relative salience. Right: N2pc and Ptc amplitudes as a function of target-decoy separation and relative salience. Note that N2pc amplitude increases with increasing target-decoy separation and N2pc amplitude increases when the decoy is relatively more salient than the target. Ptc amplitude is more positive when the target and decoy are near each other and did not vary with the relative salience manipulation. Error bars are standard error. (b) Scalp distribution of the N2pc. (c) Scalp distribution of the Ptc.

The amplitude of the N2pc component is larger when the target and decoy are far apart and smaller when the target and decoy are near each other. In addition, N2pc amplitude is larger when the decoy is relatively salient compared to when the target is relatively salient (see **Figures 6** **&** **7a**). **Figure 7b** shows the scalp distributions of the N2pc component. The scalp distributions show the N2pc as a negativity at posterior electrodes sites contralateral to the attended items. Moreover, the scalp distributions show that the N2pc component varies with target-decoy separation and with the relative salience of the target and decoy.

The N2pc data showed a main effect of target-decoy separation at P7/8, *F*(1,12) = 33.61, *p<*0.05, η_p_^2^ = 0.74, and PO3/4, *F*(1,12) = 15.57, *p<*0.01, η_p_^2^ = 0.57, indicating that N2pc amplitude was larger when the target and decoy were far apart compared to when they were near each other. The N2pc data showed a main effect of relative salience at P7/8, *F*(1,12) = 6.37, *p<*0.05, η_p_^2^ = 0.35, and a marginally significant main effect of relative salience at PO3/4, *F*(1,12) = 4.63, *p = *0.052, η_p_^2^ = 0.28, indicating that N2pc amplitude was larger when the decoy was relatively salient compared to when the target was relatively salient. The interaction between target-decoy separation and relative salience was not significant at electrodes P7/8 or PO3/4 (*F*s<1). These results confirmed our hypothesis that N2pc amplitude is affected by spatially-mediated competition and by the relative salience of the target and decoy.

#### Ptc Component

The Ptc component varies only with target-decoy separation and not with the relative salience of the target and decoy (see **Figures 6** **and** **7a**). **Figure 7c** shows the scalp distributions of the Ptc component. The scalp distributions show the Ptc as a positivity at posterior-temporal electrodes sites contralateral to the attended items. The Ptc data revealed a main effect of target-decoy separation at electrodes P7/8, *F*(1,12) = 7.57, *p*<0.05, η_p_^2^ = 0.39, and electrodes PO3/4, *F*(1,12) = 5.41, *p*<0.05, η_p_^2^ = 0.31, indicating that Ptc amplitude was more positive when the target and decoy were near each other compared to when they were far apart. The main effect of relative salience was not statistically significant at P7/8, *F*(1,12)<1, η_p_^2^ = 0.06, or PO3/4, *F*(1,12)<1, η_p_^2^ = 0.00. The interaction between target-decoy separation and relative salience was not statistically significant at P7/8, *F*(1,12)<1, η_p_^2^ = 0.02, or PO3/4, *F*(1,12)<1, η_p_^2^ = 0.00. These results indicate that the Ptc component was sensitive to target-decoy separation but was not statistically sensitive to the relative salience manipulation.

## Discussion

In two experiments, we examined electrophysiological indices of biased competition. The results of Experiment 1 show a clear dissociation between the N2pc and Ptc components. Participants performed a same/different task which forced them to process two letters at once and decide if they were the same or different. N2pc amplitude varied with the distance between the two targets such that N2pc was largest when the two targets were distant and was reduced as the two targets were moved closer together. In contrast, Ptc amplitude did not vary with the distance between the two targets. In this experiment, participants needed to identify both targets to perform the task. The participants did not need to bias the competition towards one target or the other and thus Ptc amplitude did not vary with separation between the two targets. These results are consistent with the interpretation that, while the N2pc indexes spatially-mediated competition, the Ptc indexes additional processing that helps to individuate or isolate one of the objects and that this additional processing was not necessary for this task.

In Experiment 2, we hypothesized that the N2pc component would be sensitive to both the target-decoy separation and relative salience manipulation while the Ptc component would only be sensitive to the target-decoy separation manipulation. Following our predictions, N2pc amplitude was larger when the target and decoy were far apart and smaller when they were near each other. Moreover, N2pc amplitude was larger when the decoy was relatively salient compared to when the target was relatively salient. When the decoy was relatively salient, it is likely that participants selected the salient decoy first and then shifted to the target [14]–[15]. This was reflected in larger N2pc amplitude indicating selection of the salient decoy with a subsequent shift to the target. In contrast, when the target was more salient, the target was selected initially and N2pc amplitude was smaller because there was no additional shift of attention necessary. The Ptc results also followed our predictions such that Ptc amplitude was more positive when the target and decoy were near each other and Ptc amplitude was less positive when the target and decoy were far apart. In contrast, Ptc amplitude did not vary with the relative salience manipulation. When the target and decoy are near each other, they compete for representation in extrastriate cortex. Once the target is identified, there is additional processing that individuates or isolates the target and this is reflected in a larger Ptc amplitude.

According to the biased competition theory, when multiple items are present in the visual field, they must compete for representation in the visual system [1] and this competition increases as the stimuli get closer together [18]–[21]. Moreover, this competition takes the form of mutual suppression between the items [18]–[21]. In both our experiments, N2pc amplitude was reduced when the two items were close together as opposed to far apart which is consistent with the idea that the reduced N2pc amplitude reflects spatially-mediated competition between the two items. As the two items are presented closer together, mutually suppressive competitive interactions increase and this results in decreased N2pc amplitude. Because the N2pc component is thought to reflect target selection processes in the extrastriate cortex [12], it is possible that decreased N2pc amplitude reflects degraded selection of the target in extrastriate areas due to competition between the items for representation in these brain areas. This interpretation corresponds with recent fMRI results that suggest competition for representation interferes with the ability to bias processing towards multiple attended objects in extrastriate area V4 and this results in degraded target selection [22].

These N2pc results are seemingly inconsistent with other findings reported in the literature. For example, Luck and colleagues [9] compared the N2pc evoked by a single target to the N2pc evoked by a target flanked by a nearby distractor. They found that N2pc amplitude increased when the target was presented with the distractor compared to when the target was presented alone. We have shown the apparently contradictory finding that N2pc amplitude is reduced as two attentionally salient objects are brought close together in space. However, we believe this discrepancy can be resolved. It is likely that the difference in N2pc amplitude shown by Luck and colleagues [9] was due to the difference in the number of stimuli present in the two conditions. When a single target is present, certain populations of extrastriate neurons are active representing the target. When the target and distractor are present, more neurons are active; the neurons that represent the target, the distractor, and the neurons being competed for by the target and distractor are all active and this leads to a larger N2pc. If the distance between the target and distractor was increased, the proportion of neurons independently representing the target and distractor would increase resulting in an even larger N2pc. To test this idea, a future study would need to compare a single target condition to conditions with a nearby and far away distractor.

Mazza, Turatto, and Caramazza [10] pointed out this confound evident in Luck and colleagues [9] manipulation. Specifically, Luck and colleagues [9] confounded distractor numerosity with distractor proximity. Mazza and colleagues [10] held the number of distractors constant while varying the distance between a target and these distractors. They found that N2pc amplitude did not vary with the distance between the target and distractors. The lack of N2pc modulation found by Mazza and colleagues [10] is possibly due to the magnitude of the manipulation of separation between the target and the distractors. The separation between target and distractors only varied 1° between conditions. We have shown that when the separation manipulation is larger (a difference of at least 4°), N2pc amplitude does modulate with the distance between a target and nearby distractor.

We also provided evidence of a subsequent component, termed the Ptc, as a component that potentially indexes additional processing that individuates or isolates one of the objects (i.e., the target) after it is identified. The Ptc component is so named because it is a *positivity* that was found to be distributed more towards the *temporal* electrodes (compared to the more posterior N2pc component) *contralateral* to attended objects [13]. In the target-decoy paradigm of Experiment 2, Ptc amplitude was most positive when the target and decoy were adjacent and was less positive as the distance between the two attended items increased which replicates our previous results [13]. In contrast, when both targets need to be processed as in the same-different task of Experiment 1, Ptc amplitude did not vary with the distance between the two targets. These results suggest that Ptc amplitude may be influenced by the amount of additional processing that is necessary to overcome the spatially-mediated competition for representation. We have argued that, because the Ptc occurs after the N2pc component, the Ptc probably reflects processing subsequent to target identification. Thus it is plausible that the Ptc component reflects processes used to individuate or isolate the target once it is identified. This additional processing is necessary when the target and decoy are near each other and thus competing for representation. However, when the two objects are farther apart, there is minimal competition for representation which reduces the need for additional processes to individuate or isolate the target.

It must be noted that we have only shown a single dissociation between the N2pc and Ptc components. Specifically, we manipulated N2pc amplitude while Ptc amplitude remained unaffected but we have not shown that Ptc amplitude can be manipulated while N2pc amplitude remains unaffected. Thus, without a double dissociation, it still remains possible that activity in the Ptc interval merely reflects late N2pc activity. Another possibility is that the Ptc component is actually a subcomponent of the N2pc called the Pd component. The Pd is a positive component distributed over posterior scalp regions contralateral to a distractor and may reflect suppression of task-irrelevant distractors [23]–[24]. Due to design limitations of the current study, it is not possible to determine whether the Ptc reflect target processing, distractor processing, or both. Future studies should isolate target related processing from distractor related processing to help determine the relationship between the Ptc and Pd components.

### Summary

In two experiments, we examined ERPs that dissociate the effects of salience and space in biased competition for visual representation. In Experiment 1, we used a same-different task to dissociate the N2pc component from the Ptc component. Participants responded to two targets by indicating whether they were same or different letters. The distance between the two targets was systematically manipulated. Results indicate that the N2pc component varied with target-target separation but the Ptc did not. In Experiment 2, participants responded to the orientation of a target while ignoring a decoy. The distance between the target and decoy and the relative salience of the target and decoy were manipulated. Results indicate that the N2pc was sensitive to both the distance and relative salience manipulations while the Ptc was modulated by only the distance manipulation. Taken together, these results are consistent with the idea that the reduction in N2pc amplitude reflects degraded target selection due to spatially-mediated competition while the Ptc indexes additional processes used to individuate or isolate the target once it is identified.
